# Quarantine1Read at the 37th annual meeting of the Medical Association of Central New York, held at Rochester N. Y., October 18, 1904.

**Published:** 1905-05

**Authors:** F. H. Sawers

**Affiliations:** Rochester, N. Y.


### Quarantine.[1]

BY F. H. SAWERS, M. B., Rochester, N. Y.

VIGILANCE and measures for the public good are highly commendable and need no defense. There are some matters, however, the attending physician is well fitted to supervise and his place should not be usurped nor interfered with by those in power without some very valid reason and, surely, never without ample opportunity being given him to be heard.

The inadequate remarks in this paper are meant to bring out some thoughts along this line, as well as something in reference to the protection the citizen deserves. They are not intended to offend, the desire being that they may bear some good fruit.

What great benefits quarantine has brought to the people of our day. Compare the manner of safeguarding the community now to the barbaric times of killing the victims and burning their effects. How much less interference with commercial affairs scientific and advanced methods, as practised now, afford without the terrifying restrictions of early times.

The value of quarantine properly and carefully applied meets with little opposition at this time and its efficiency, although imperfect, has numerous instances to attest its great utility. It would be difficult indeed to compute the saving of that most precious thing, life, and the preserving of health, so desirable for the individual as well as the nation, which quarantine measures have effected; and to accomplish something worth while in this way the few must often, of course, suffer inconvenience for the good of the many. What is of such vital concern to all meets with a ready response from the medical profession in the fullest sense to aid in forming just and effective laws for the framing of which their education and experience so well fit them.

It is not my purpose in this short article to have included in it any extended or specific details relating to the methods of quarantine, but rather to express a few thoughts bearing on the subject of the seriousness of jeopardising the citizens prerogative and that of his attendant, whoever he may be. Possibly it can best be done by illustration.

A physician on board an ocean liner was sick with tonsillitis

---

1. Read at the 37th annual meeting of the Medical Association of Central New York, held at Rochester N. Y., October 18, 1904.

and at the same time a simple ecthymatous eruption made its appearance on his body, which was diagnosticated by the ship's doctor as smallpox. He said he would be compelled to isolate the case; but the physician passenger disagreed with him and maintained the former diagnosis was the correct one, demurring at the same time to the order of removal to quarantine. Happily the order was not put into effect. One might say that the authority of the ship's doctor warranted the isolation of the patient, but we would all agree that the doctor's action was not only commendable, but just. What inconvenience and fear his judicious course averted! In a couple of days the physician, whose indisposition had disappeared, rejoined the ship's doctor on deck, with no less friendship.

How careful we ought to be when a mistake may mean so much to life and property. We are all so liable to err; indeed, it is the lot of man, so frail yet so strong, that when he sees his error and admits it, he at once becomes the conqueror.

During a clinical test, Mr. Sydney Jones, chief surgeon of Saint Thomas's Hospital, London, and one of the foremost men of that great city, said to a student whom he was examining, "You are careful enough in your work in this case, but I cannot understand how you can make out such symptoms and reach the conclusion you do. Let us review it." This concession by Mr. Jones was of great importance to the student and was much appreciated. It is needless to add that the impressive lesson taught by this noble man has probably never been forgotten by the student, nor his admonition, "admit your errors, be just, retain your selfrespect, and the good opinion of your adversary."

Not long ago, a child a year and a half old, with slight fever, a light reddish-brown papular eruption, not continuous, disappearing on the second day with little desquamation, enlarged posterior cervical glands, came under my observation; also a brother a year older, sick a day or two before; no eruption when seen, posterior cervical glands enlarged. The mother of these children was indisposed for a few days, complaining of sore throat, but not confined to bed, nor unable to attend to her usual duties; when seen, there was no eruption, no objective signs of sore throat, no desquamation, but an enlarged posterior cervical gland. These cases were diagnosticated as German measles.

Ten days later the mother's sister, who lived about a mile away and who had called to see these relatives, was sent by the health department to the scarlet fever pavilion, and the house of the former was placarded with a scarlet fever sign. Was this not a violation of the citizen's prerogative? But we were told that the mother's hands were peeling. True, due to turpentine which a kind neighbor had recommended to ease pain. Although the subject matter was thoroughly presented to the attention of the authorities repeatedly, they still maintained there was no reason for a change of mind and vouchsafed the information, that when cases were not reported prosecution could be instituted.

Although assumed to be a reportable disease, German measles is not incorporated in the ordinance, and therefore it is not necessary to report it; neither is it just to one's patients, nor advantageous to the interest of the community at large to do so. By magnifying mild diseases, we lessen the value of otherwise effective measures to guard against the dangerous ones. By unwarrantable errors, the people are put to great inconvenience, and the most important office in the community is ridiculed and feared, instead of being respected and trusted; and the danger to the community from concealed diseases which are of a serious nature, are very much increased.

As a duty to the public which must needs look to the medical profession for betterment, along lines pertaining to public health, let us bend our energies to secure capable, just, conscientious and fearless officials, who are unhampered by partisan claims; men who will trust the public, and in turn be trusted; always solicitous for the liberty and safety of the individual, while acting with a firm, yet kind hand, for the welfare of all.

548 Lake Avenue.

